# High activity of Ag-doped Cd_0.1_Zn_0.9_S photocatalyst prepared by the hydrothermal method for hydrogen production under visible-light irradiation

**DOI:** 10.3762/bjnano.5.69

**Published:** 2014-05-07

**Authors:** Leny Yuliati, Melody Kimi, Mustaffa Shamsuddin

**Affiliations:** 1 Ibnu Sina Institute for Fundamental Science Studies, Universiti Teknologi Malaysia, 81310 UTM Johor Bahru, Johor, Malaysia; 2 Department of Chemistry, Faculty of Science, Universiti Teknologi Malaysia, 81310 UTM Johor Bahru, Johor, Malaysia; 3 Centre for Pre-University Studies, Universiti Malaysia Sarawak, 94300 Kota Samarahan, Sarawak, Malaysia

**Keywords:** Ag doping, Cd_0.1_Zn_0.9_S, hydrogen production, hydrothermal, visible light

## Abstract

**Background:** The hydrothermal method was used as a new approach to prepare a series of Ag-doped Cd_0.1_Zn_0.9_S photocatalysts. The effect of Ag doping on the properties and photocatalytic activity of Cd_0.1_Zn_0.9_S was studied for the hydrogen production from water reduction under visible light irradiation.

**Results:** Compared to the series prepared by the co-precipitation method, samples prepared by the hydrothermal method performed with a better photocatalytic activity. The sample with the optimum amount of Ag doping showed the highest hydrogen production rate of 3.91 mmol/h, which was 1.7 times higher than that of undoped Cd_0.1_Zn_0.9_S. With the Ag doping, a red shift in the optical response was observed, leading to a larger portion of the visible light absorption than that of without doping. In addition to the larger absorption in the visible-light region, the increase in photocatalytic activity of samples with Ag doping may also come from the Ag species facilitating electron–hole separation.

**Conclusion:** This study demonstrated that Ag doping is a promising way to enhance the activity of Cd_0.1_Zn_0.9_S photocatalyst.

## Introduction

The development of clean and renewable hydrogen energy through a sustainable production process is still a big issue to be addressed. Solar energy is a very attractive option as it is the most abundant energy. The conversion of solar energy to chemical energy by photocatalytic processes, such as photocatalytic water reduction in the presence of semiconductor photocatalysts, would be an opportunity to produce clean hydrogen energy. Recently, special attention has been paid to the use of visible light-driven photocatalysts [1–4]. One of the promising photocatalysts is Cd_1−_*_x_*Zn*_x_*S solid solution [5–8]. The successful formation of a solid solution of ZnS and CdS resulted in an absorption shift of ZnS to the visible-light range, while maintaining the high conduction band energy required for hydrogen production. However, in order to utilize solar energy in the future, a further red shift to a range of even longer wavelengths is still highly desired.

The modification of Cd_1−_*_x_*Zn*_x_*S photocatalyst with metal ions, such as Cu [9–13], Ni [14–15], Sn [16], and Sr [17] has been a good attempt to increase the visible-light absorption of the Cd_1−_*_x_*Zn*_x_*S photocatalyst. The use of Ag species as a good dopant for various types of photocatalysts has been also reported [18–20], including its use to modify Cd_1−_*_x_*Zn*_x_*S [21–23]. Cd_1−_*_x_*Zn*_x_*S modified by Ag_2_S was reported to show activity for hydrogen production from water [21] and hydrogen sulfide [22]. On the other hand, the properties of Ag^+^-doped Cd_1−_*_x_*Zn*_x_*S have been investigated by spectroscopic and photochemical studies [23]. It was proposed that the Ag^+^ might act as a hole trapping site. Since the electron–hole recombination rate may increase as a result of defect sites created by the doping element, reducing electron–hole recombination and promoting interfacial charge transfer should be optimized in order to improve the efficiency of the photocatalysts.

The most widely used method to prepare Ag-doped Cd_1−_*_x_*Zn*_x_*S is the co-precipitation method [21]. However, the co-precipitation method usually produces materials with low crystallinity. Since high crystallinity is beneficial for photocatalytic hydrogen production [1–4], it is worth to further investigate an alternative method to prepare the Ag-doped Cd_1−_*_x_*Zn*_x_*S with high crystallinity. It has been reported that compared to the co-precipitation method, the hydrothermal method produced sulfide photocatalysts with better crystallinity, which gave higher activity for hydrogen production [9,16]. In the present work, the Ag(*x*)-doped Cd_0.1_Zn_0.9_S samples were prepared by both hydrothermal and co-precipitation methods. The superior activity of Ag(*x*)-doped Cd_0.1_Zn_0.9_S prepared by hydrothermal method is discussed.

## Results and Discussion

**Figure 1:**
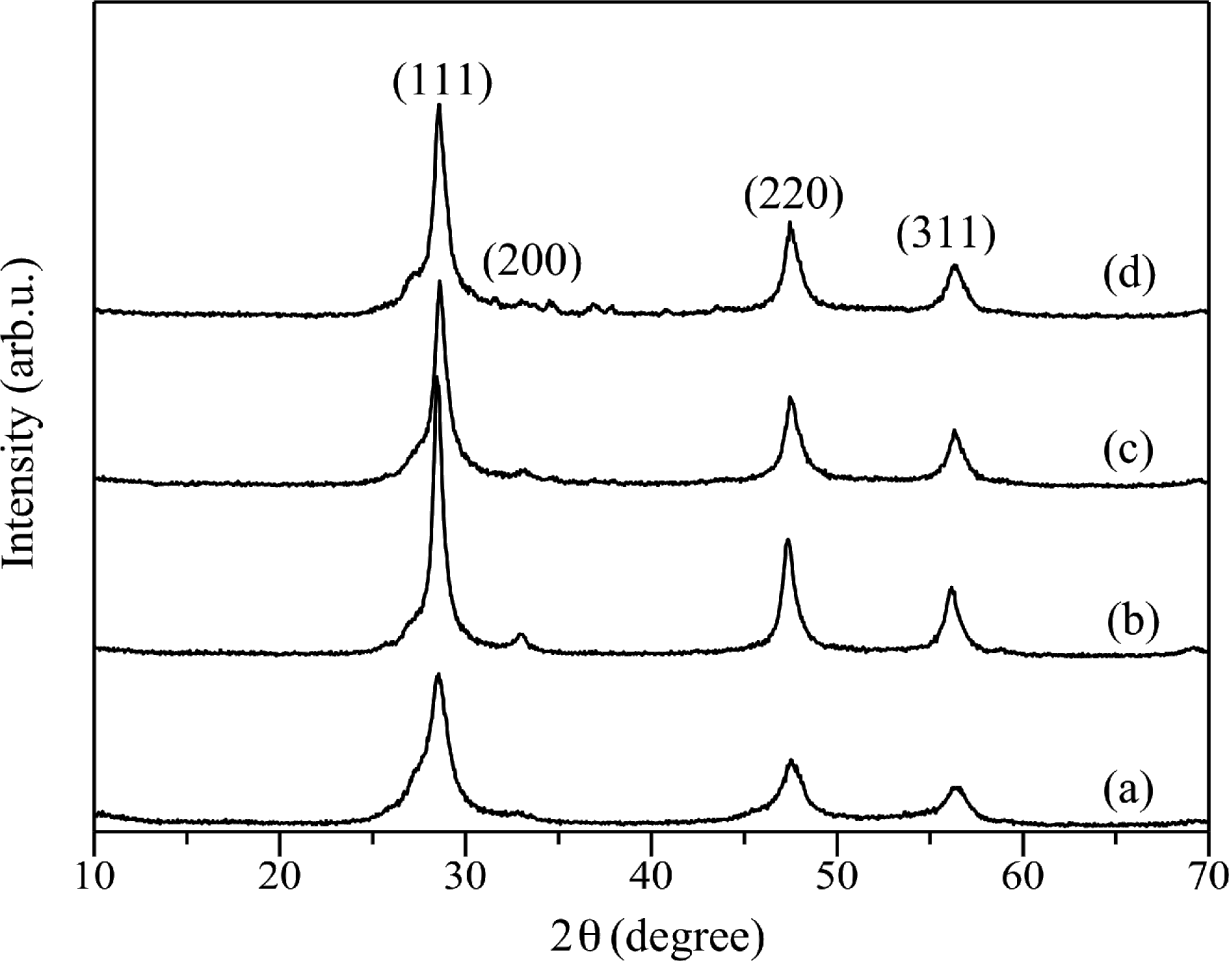
XRD patterns of (a) Cd_0.1_Zn_0.9_S, (b) Ag(0.01)-doped Cd_0.1_Zn_0.9_S, (c) Ag(0.03)-doped Cd_0.1_Zn_0.9_S, and (d) Ag(0.05)-doped Cd_0.1_Zn_0.9_S prepared by the hydrothermal method.

Figure 1 shows the X-ray diffraction (XRD) patterns of Cd_0.1_Zn_0.9_S and Ag(*x*)-doped Cd_0.1_Zn_0.9_S samples prepared by using the hydrothermal method. The diffraction peaks for all samples, except for Ag(0.05)-doped Cd_0.1_Zn_0.9_S, were in good agreement with the diffraction peaks of ZnS cubic zinc-blende phase (JCPDS No. 772100) with major diffraction peaks at 2θ of 28.6, 32.5, 47.6 and 56.3°, corresponding to the (111), (200), (220) and (311) planes respectively. On the other hand, in addition to the cubic zinc blende phase, the Ag(0.05)-doped Cd_0.1_Zn_0.9_S also showed the presence of small diffraction peaks of the hexagonal phase at 2θ of ca. 27 and 31° (Figure 1d). A similar phenomenon was also reported when Cu was used as a dopant [9]. There are no diffraction peaks corresponding to Ag or other crystal phases. This could be due to the fact that the content of Ag might be too small to be detected or Ag was well dispersed in Cd_0.1_Zn_0.9_S. This result also indicated that no detectable impurity phases existed in the prepared samples. The small amount of Ag dopant increased remarkably the intensity of the diffraction peaks compared to the undoped Cd_0.1_Zn_0.9_S (Figure 1a,b), which suggests that a small amount of Ag might induce the crystal growth. However, further increase of the Ag dopant did not further increase the intensity of the diffraction peaks. With increasing amount of Ag dopant, the peaks became slightly broader (Figure 1b–d) since Ag might be clustered and in turn gave a slightly increased disorder. As the diffraction peaks were only slightly shifted to higher values of 2θ with increasing amount of Ag, it can be suggested that Ag could be doped into the lattice without causing much crystal distortion.

**Figure 2:**
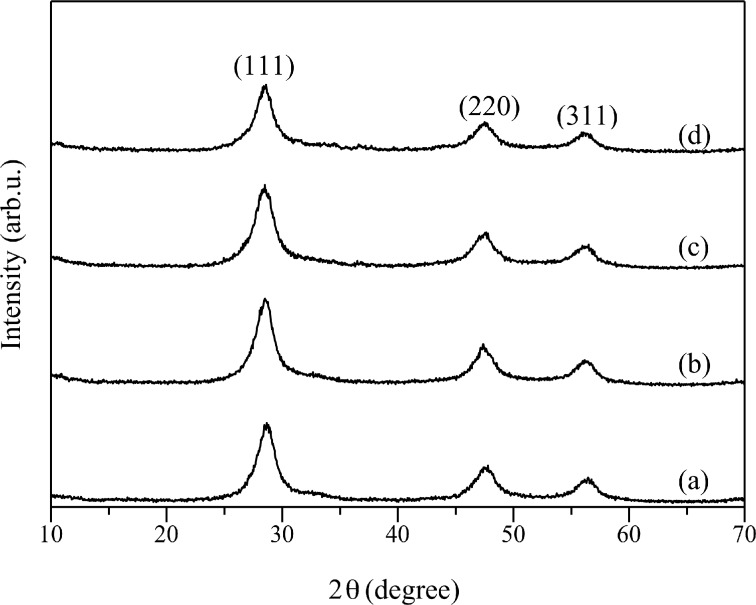
XRD patterns of (a) Cd_0.1_Zn_0.9_S, (b) Ag(0.01)-doped Cd_0.1_Zn_0.9_S, (c) Ag(0.03)-doped Cd_0.1_Zn_0.9_S, and (d) Ag(0.05)-doped Cd_0.1_Zn_0.9_S prepared by the co-precipitation method.

The XRD patterns for Cd_0.1_Zn_0.9_S and Ag(*x*)-doped Cd_0.1_Zn_0.9_S samples prepared by the co-precipitation method are shown in Figure 2. For all samples, only diffraction peaks of ZnS cubic zinc-blende phase could be observed and no other phases could be detected. Different from the series prepared by hydrothermal method, there was no obvious change in the intensities of diffraction peaks after Ag was doped into the Cd_0.1_Zn_0.9_S. Broadening and shifting of the diffraction peaks were not observed, suggesting that Ag might not be doped inside but existed on the surface of Cd_0.1_Zn_0.9_S. Samples prepared by the co-precipitation method showed less intense and broader diffraction peaks than those prepared by the hydrothermal method, suggesting the less crystallinity and/or less crystallite size. This result was reasonable since co-precipitation method did not involve crystal growth by heating process.

**Figure 3:**
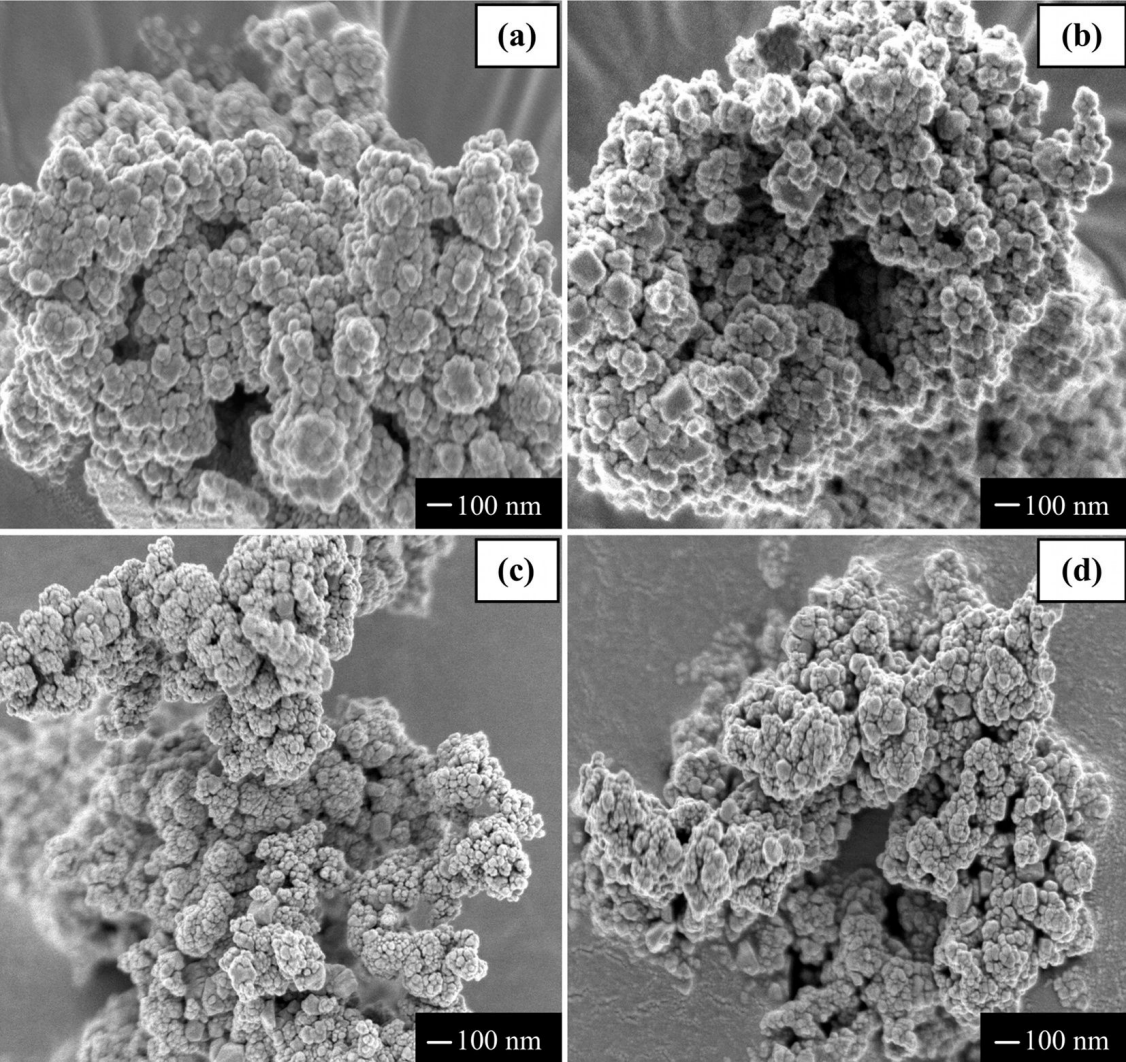
FESEM images of (a) Cd_0.1_Zn_0.9_S, (b) Ag(0.01)-doped Cd_0.1_Zn_0.9_S, (c) Ag(0.03)-doped Cd_0.1_Zn_0.9_S (d) Ag(0.05)-doped Cd_0.1_Zn_0.9_S prepared by the hydrothermal method.

**Figure 4:**
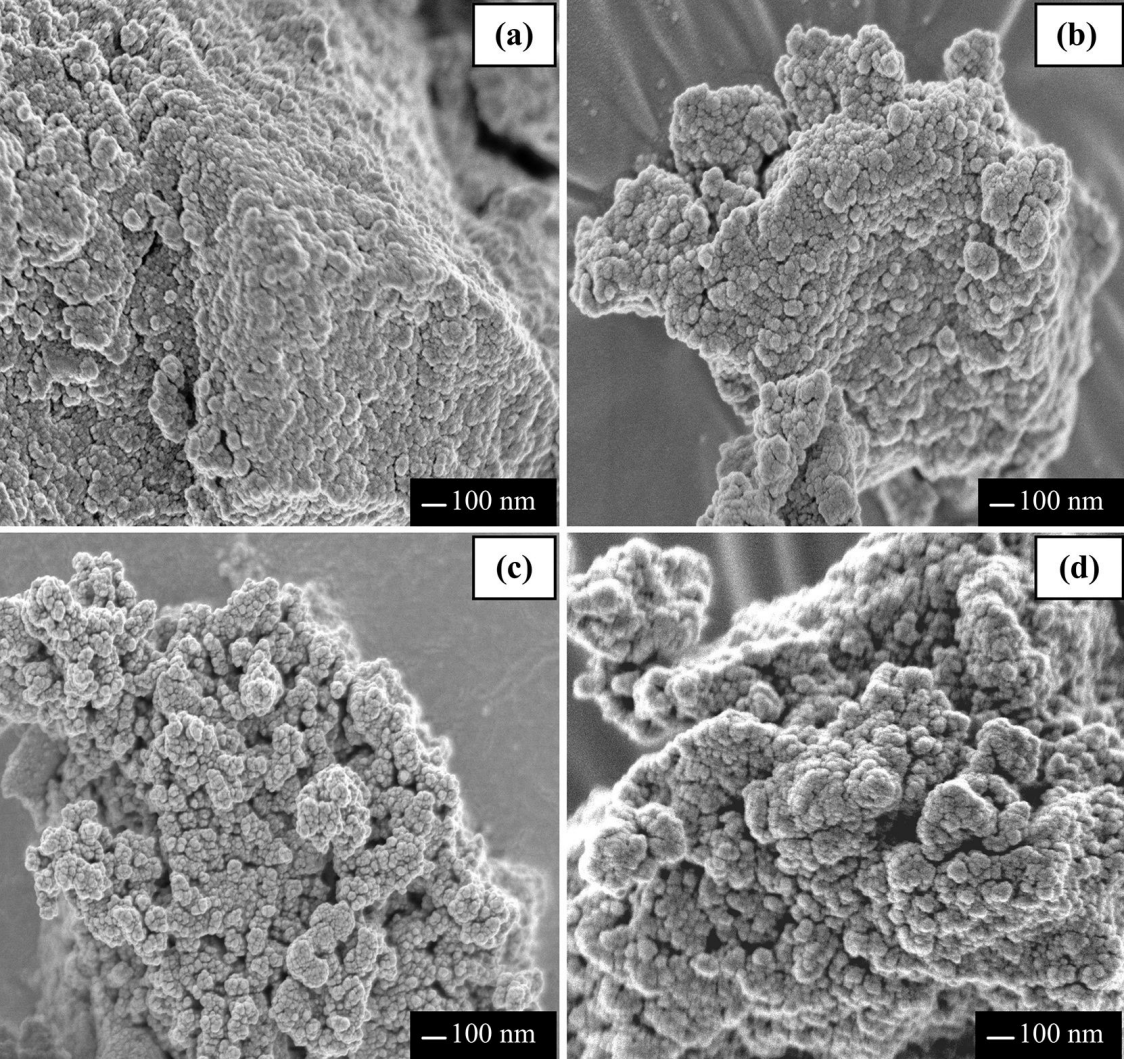
FESEM images of (a) Cd_0.1_Zn_0.9_S, (b) Ag(0.01)-doped Cd_0.1_Zn_0.9_S, (c) Ag(0.03)-doped Cd_0.1_Zn_0.9_S (d) Ag(0.05)-doped Cd_0.1_Zn_0.9_S prepared by the co-precipitation method.

Figure 3 and Figure 4 show field emission scanning electron microscopy (FESEM) images of Cd_0.1_Zn_0.9_S and Ag(*x*)-doped Cd_0.1_Zn_0.9_S samples prepared by hydrothermal and co-precipitation methods, respectively. For samples prepared by the hydrothermal method, all samples have a spherical shape with particle sizes in the range of 20–120 nm that are further agglomerated into bigger particles with no uniform size. The samples prepared by the co-precipitation method also have spherical shapes with slightly lower particle sizes in the range of 10–70 nm. For all samples, the morphology of Cd_0.1_Zn_0.9_S was not affected by the added Ag.

**Figure 5:**
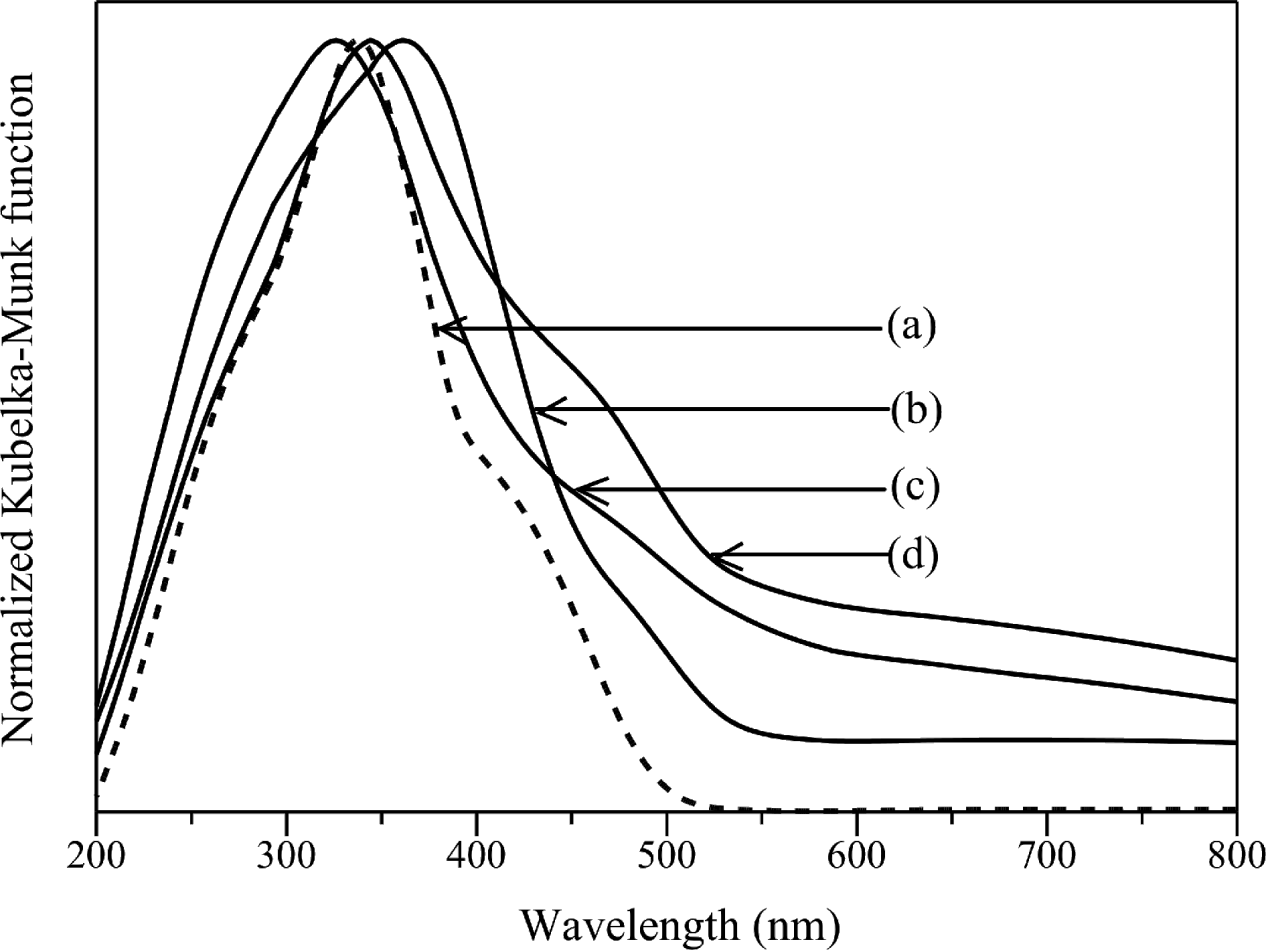
DR UV–visible spectra of (a) Cd_0.1_Zn_0.9_S, (b) Ag(0.01)-doped Cd_0.1_Zn_0.9_S, (c) Ag(0.03)-doped Cd_0.1_Zn_0.9_S (d) Ag(0.05)-doped Cd_0.1_Zn_0.9_S prepared by the hydrothermal method.

Figure 5 shows the diffuse reflectance UV–visible (DR UV–vis) spectra of samples prepared by the hydrothermal method. The Cd_0.1_Zn_0.9_S showed a shoulder peak around 400–500 nm (Figure 5a), similar to previous studies [9,16]. The addition of Ag shifted the absorption edge toward longer wavelengths, suggesting the formation of Ag-doped Cd_0.1_Zn_0.9_S samples. The values of the band gap energy for the samples are listed in Table 1. The band gap energy was determined by taking the intersection between the linear tangent line with the *x*-axis from a plot of F(%R)*h*ν^1/^*^n^* versus hν, in which F(%R) is the Kubelka–Munk function, *h* is Planck’s constant, ν is the frequency of vibration, and *n* is 1/2 for a direct allowed transition. As shown in Table 1, the addition of a small amount of Ag decreased the band gap energy of the Cd_0.1_Zn_0.9_S samples (Table 1, entries 1 and 2). A further increase of Ag did not give a monotonous decrease in the band gap energy, even though these samples still showed lower band gap energy than the Cd_0.1_Zn_0.9_S sample (Table 1, entries 3 and 4).

**Table 1:** Band gap energy values for Cd_0.1_Zn_0.9_S and Ag-doped Cd_0.1_Zn_0.9_S samples.

entry	sample	band gap energy/eV	preparation method

1	Cd_0.1_Zn_0.9_S	3.11	hydrothermal
2	Ag(0.01)-doped Cd_0.1_Zn_0.9_S	2.81	
3	Ag(0.03)-doped Cd_0.1_Zn_0.9_S	3.01	
4	Ag(0.05)-doped Cd_0.1_Zn_0.9_S	2.88	

5	Cd_0.1_Zn_0.9_S	2.94	co-precipitation
6	Ag(0.01)-doped Cd_0.1_Zn_0.9_S	3.06	
7	Ag(0.03)-doped Cd_0.1_Zn_0.9_S	2.99	
8	Ag(0.05)-doped Cd_0.1_Zn_0.9_S	2.66	

In addition to the shifted absorption edge and the decreased band gap energy, Ag doping caused an absorption tail in the range of 600–800 nm. The absorption in this area has been assigned to the formation of Ag_2_S [21,24]. Even though the Ag_2_S formation could not be detected by XRD owing to the low sensitivity, it can be suggested that the added Ag was not completely doped in the Cd_0.1_Zn_0.9_S. The addition of Ag also caused a new absorption appearing at about 295 nm for Ag(0.01)-doped Cd_0.1_Zn_0.9_S and Ag(0.03)-doped Cd_0.1_Zn_0.9_S (Figure 5b,c), corresponding to the presence of Ag metal nanoparticles [25] that can be formed catalytically through the reduction of Ag^+^ by the Ag_2_S as the catalyst [26]. On the other hand, even though Ag(0.05)-doped Cd_0.1_Zn_0.9_S did not show such an absorption at 295 nm (Figure 5d), its maximum peak was shifted to longer wavelengths, which might occur due to the increase in the particle size of the formed Ag metal [26]. Even though the mechanism could not be revealed in this paper, these results showed that the addition of Ag was not only simply doped into the Cd_0.1_Zn_0.9_S, but also was used to form other Ag species such as Ag metal and Ag_2_S. The formation of the later species was obviously increased with the increase of the added Ag amount. Various possible formations of Ag species mentioned above led to the non-monotonous red shift and non-monotonous decrease in the band gap energy with the increase of Ag doping.

**Figure 6:**
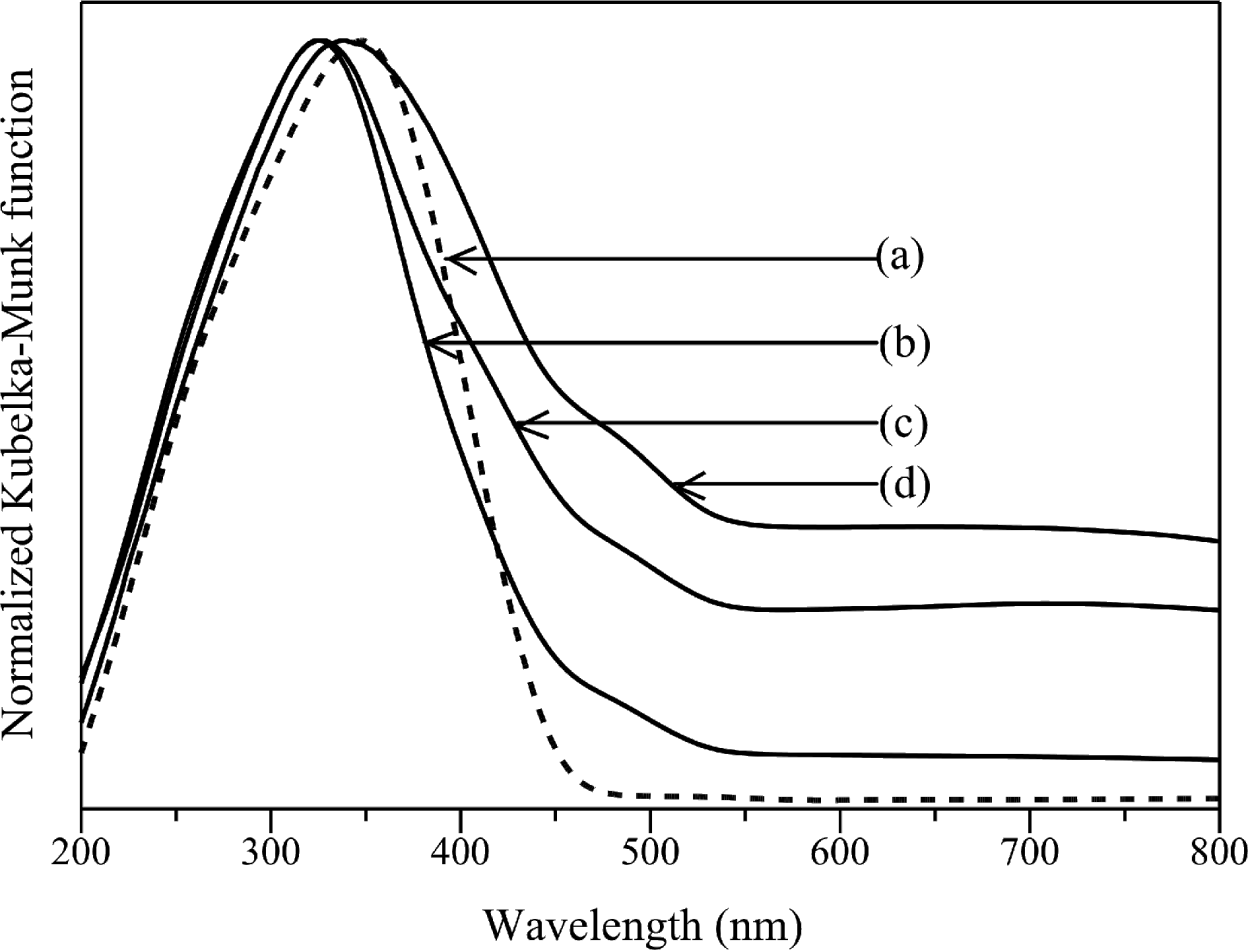
DR UV–visible spectra of (a) Cd_0.1_Zn_0.9_S, (b) Ag(0.01)-doped Cd_0.1_Zn_0.9_S, (c) Ag(0.03)-doped Cd_0.1_Zn_0.9_S (d) Ag(0.05)-doped Cd_0.1_Zn_0.9_S prepared by the co-precipitation method.

The absorption spectra of samples synthesized by co-precipitation method are shown in Figure 6. The Cd_0.1_Zn_0.9_S sample showed a smooth absorption edge around 450 nm (Figure 6a). The addition of Ag shifted the absorption edge toward longer wavelengths when the amount of Ag was large (Figure 6d). As shown in Table 1, a small amount of Ag slightly increased the band gap energy of Cd_0.1_Zn_0.9_S sample (Table 1, entries 5–7), while a large amount of Ag decreased the band gap energy (Table 1, entry 8). The increase in Ag doping also gave an increase in the absorption level above 600 nm due to the formation of Ag_2_S [21,24]. The formation of Ag_2_S on the samples prepared by the co-precipitation method was found to be larger than that on the samples prepared by the hydrothermal method. Even though the formation of a new absorption below 300 nm was not as clear as those observed on the samples prepared by the hydrothermal method, it could be observed that the peak maximum of Cd_0.1_Zn_0.9_S was shifted from 348 to 325–326 nm (Figure 6b,c), which would be due to formation of Ag metal on Ag(0.01)-doped Cd_0.1_Zn_0.9_S and Ag(0.03)-doped Cd_0.1_Zn_0.9_S. A further increase of the amount of added Ag caused the peak maximum to be shifted from 326 to 339 nm (Figure 6d), owing to the formation of larger particles of Ag metal, similar to the samples prepared by hydrothermal method mentioned above.

**Figure 7:**
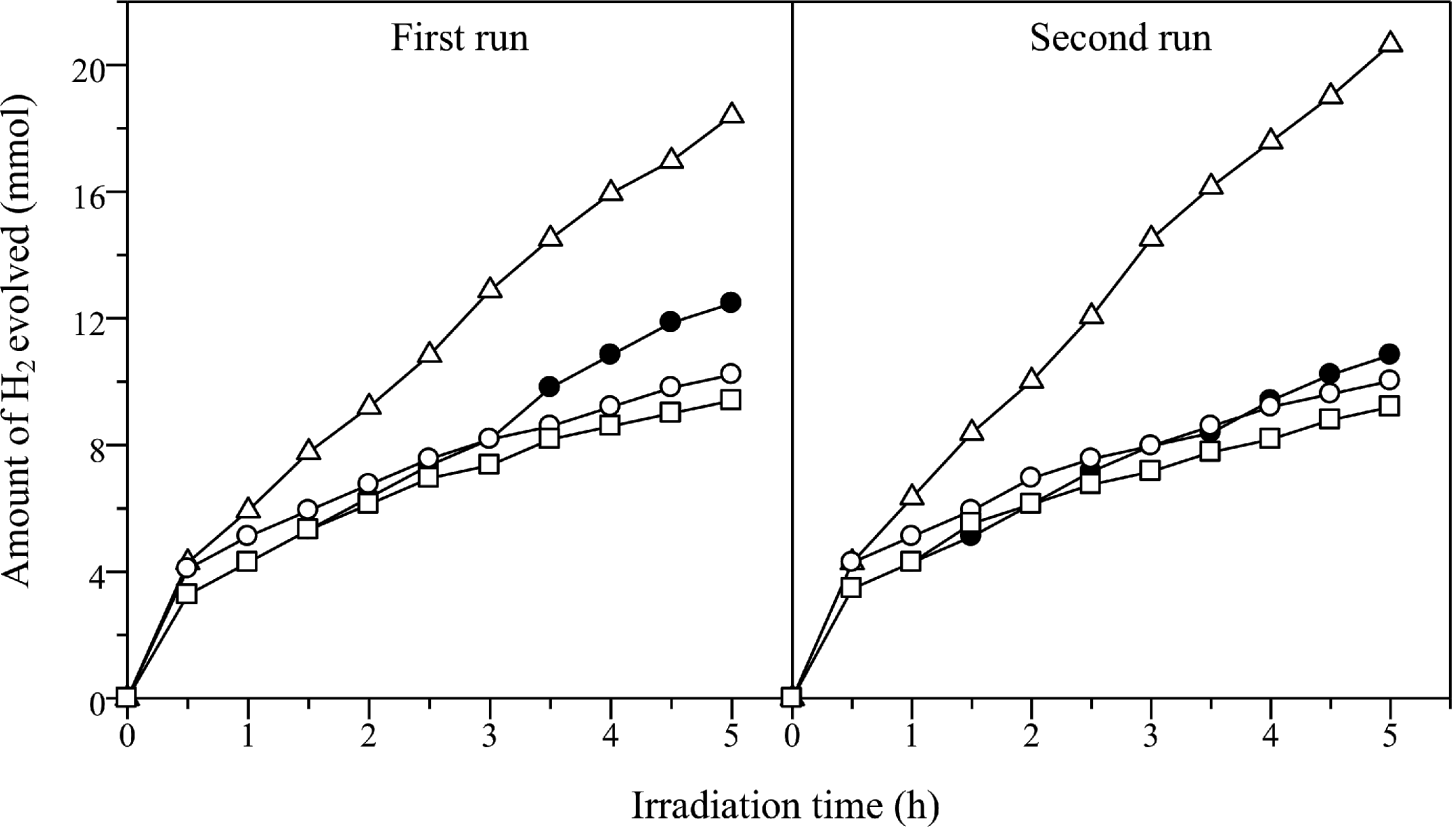
Photocatalytic hydrogen evolution on Cd_0.1_Zn_0.9_S (filled circles), Ag(0.01)-doped Cd_0.1_Zn_0.9_S (empty triangles), Ag(0.03)-doped Cd_0.1_Zn_0.9_S (empty circles), and Ag(0.05)-doped Cd_0.1_Zn_0.9_S (empty squares) prepared by the hydrothermal method under visible-light irradiation.

The photocatalytic performances of Cd_0.1_Zn_0.9_S and Ag(*x*)-doped Cd_0.1_Zn_0.9_S samples prepared by the hydrothermal method were examined for hydrogen production under visible-light irradiation as shown in Figure 7. All samples showed visible-light activity for hydrogen production. The undoped Cd_0.1_Zn_0.9_S sample produced hydrogen at a rate of 2.49 mmol/h. The Ag(0.01)-doped Cd_0.1_Zn_0.9_S showed an increased rate of hydrogen production of 3.68 mmol/h. However, unfortunately, when the amount of Ag increased, the activity was not improved. After 5 h of reaction, Ag(0.03)-doped Cd_0.1_Zn_0.9_S and Ag(0.05)-doped Cd_0.1_Zn_0.9_S even showed a lower rate than that obtained from the undoped Cd_0.1_Zn_0.9_S. The enhancement in the photocatalytic activity of Ag(0.01)-doped Cd_0.1_Zn_0.9_S might be attributed to the better crystallinity, the improved absorption in the visible-light region, as well as the presence of Ag species. Regarding the latter it has been proposed that both Ag^0^ and Ag^+^ played an important role in facilitating the charge separation and suppressing the recombination of photoexcited charge carries [18–19], while the Ag_2_S could also act as a hole-transfer catalyst [21,24] for the oxidation of sulfide ions, which in turn promoted the activity. As shown in the DR UV–vis spectra (Figure 5), the formation of Ag_2_S could not be avoided and it increased with the increase of Ag amount. However, since the activity did not increase but decreased with the increase of Ag amount, the Ag_2_S would not be the main species responsible for the high activity on Ag(0.01)-doped Cd_0.1_Zn_0.9_S. Indeed, the decrease of the activity of Ag(0.03)-doped Cd_0.1_Zn_0.9_S and Ag(*0.05*)-doped Cd_0.1_Zn_0.9_S showed that the Ag_2_S might block the incident light, reduce the reaction sites for oxidation, and thus suppress the photocatalytic reaction [24].

**Figure 8:**
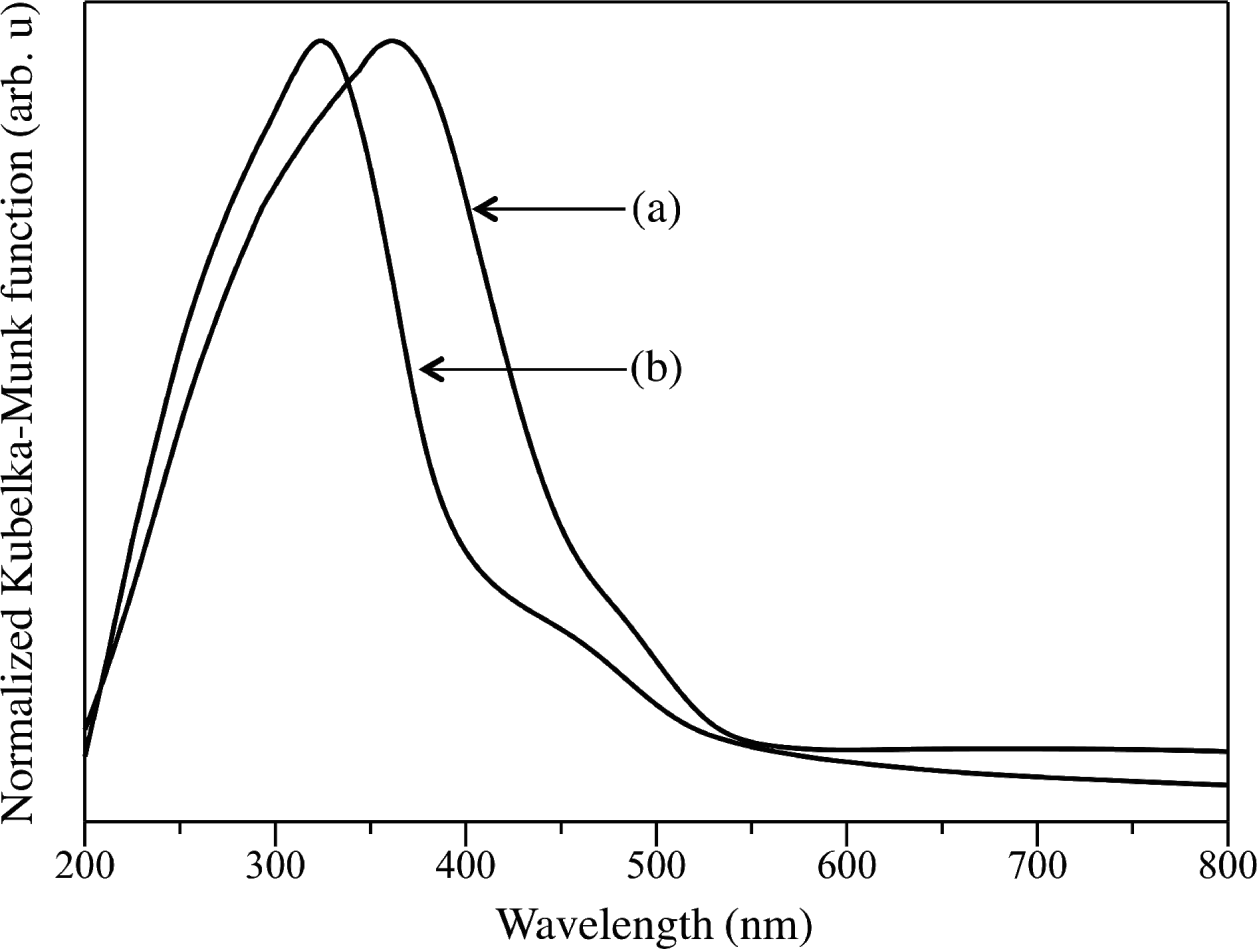
DR UV–visible spectra of (a) fresh and (b) used Ag(0.01)-doped Cd_0.1_Zn_0.9_S prepared by the hydrothermal method after second run.

The stability of the samples prepared by the hydrothermal method was investigated by reusing the samples for two consecutive runs. The photocatalytic activity results for first and second runs are also shown in Figure 7. The undoped Cd_0.1_Zn_0.9_S showed a lower photocatalytic activity in the second run due to poor stability. On the other hand, all Ag-doped Cd_0.1_Zn_0.9_S samples showed a relatively stable activity. This result suggested that the presence of Ag was important to maintain the stability of the photocatalysts. Among the samples, the Ag(0.01)-doped Cd_0.1_Zn_0.9_S sample showed the highest activity for hydrogen production. The rate for hydrogen production was slightly increased in the second run, suggesting that the Ag(0.01)-doped Cd_0.1_Zn_0.9_S sample might experience a photochemical activation process. The similar phenomenon was also reported to occur on CdS/ZnFe_2_O_4_ photocatalyst during the photocatalytic hydrogen production [27]. In order to understand the possible photochemical activation process occurred on the Ag(0.01)-doped Cd_0.1_Zn_0.9_S sample, the used sample was characterized by DR UV–visible spectroscopy. As shown in Figure 8, the absorption peak of the Ag(0.01)-doped Cd_0.1_Zn_0.9_S sample was shifted to lower wavelengths centred around 323 nm and the absorption intensity above 550 nm was decreased after the second run. These changes showed that the amount of Ag metal on the surface might be increased via photoreduction during the reaction. As a result, the photocatalytic activity of the Ag(0.01)-doped Cd_0.1_Zn_0.9_S sample increased within the reaction time. The average hydrogen production rate from both runs was determined to be 3.91 mmol/h and the value was 1.7 times better than the average rate observed on the undoped Cd_0.1_Zn_0.9_S.

**Figure 9:**
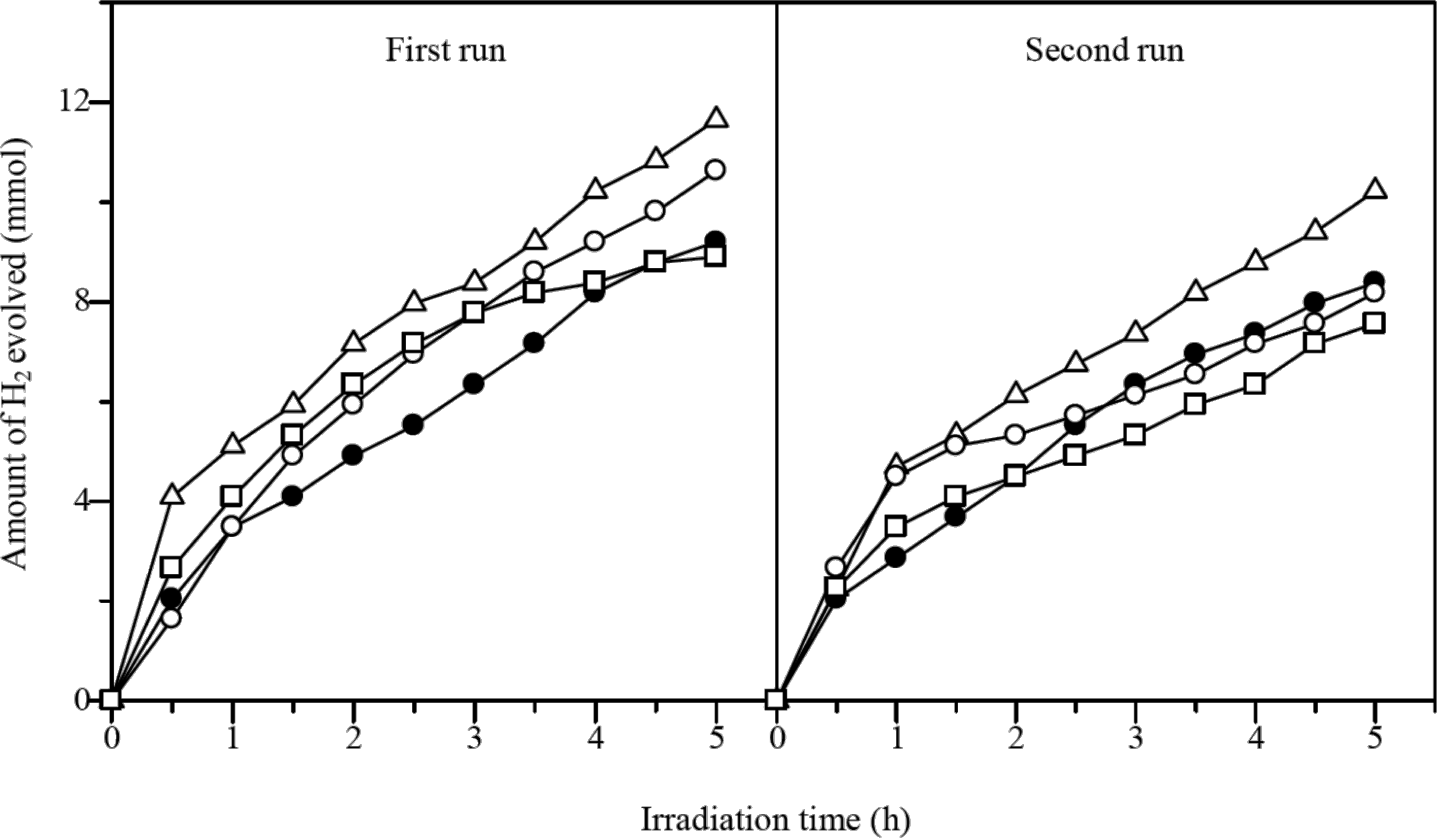
Photocatalytic hydrogen evolution on Cd_0.1_Zn_0.9_S (filled circles), Ag(0.01)-doped Cd_0.1_Zn_0.9_S (empty triangles), Ag(0.03)-doped Cd_0.1_Zn_0.9_S (empty circles), and Ag(0.05)-doped Cd_0.1_Zn_0.9_S (empty squares) prepared by the co-precipitation method under visible-light irradiation.

Figure 9 shows the photocatalytic activities of Cd_0.1_Zn_0.9_S and Ag(*x*)-doped Cd_0.1_Zn_0.9_S samples prepared by co-precipitation method. The Ag(0.01)-doped Cd_0.1_Zn_0.9_S and Ag(0.03)-doped Cd_0.1_Zn_0.9_S showed a higher activity than the undoped Cd_0.1_Zn_0.9_S. The highest photocatalytic activity was recorded for the Ag(0.01)-doped Cd_0.1_Zn_0.9_S with a hydrogen production rate of 2.19 mmol/h. As for Ag(0.05)-doped Cd_0.1_Zn_0.9_S, the activity was higher than that of the undoped compound in the first 3.5 hours, but the activity decreased over time. The samples prepared by the co-precipitation method showed less activity than those prepared by the hydrothermal method, which might be due to the lower crystallinity as discussed above. The stability of the samples was also tested for the second run. It was observed that undoped Cd_0.1_Zn_0.9_S and Ag(0.01)-doped Cd_0.1_Zn_0.9_S showed a slightly decreased photocatalytic activity as compared to activity obtained in the first run. The decrease in the activity is stronger in Ag(0.03)-doped Cd_0.1_Zn_0.9_S and Ag(0.05)-doped Cd_0.1_Zn_0.9_S. Both samples showed a lower activity than the undoped one in the second run. The Ag(*x*)-doped Cd_0.1_Zn_0.9_S samples prepared by the co-precipitation method were found to be less stable than those prepared by the hydrothermal method that might be due to the formation of more Ag_2_S and less doping of Ag.

## Conclusion

Two series of Ag-doped Cd_0.1_Zn_0.9_S samples were prepared by hydrothermal and co-precipitation methods, respectively. The Ag(0.01)-doped Cd_0.1_Zn_0.9_S prepared by the hydrothermal method showed the highest photocatalytic activity and stability under visible light irradiation with a hydrogen production rate of 3.91 mmol/h, which was 1.7 times higher than that of the undoped Cd_0.1_Zn_0.9_S. The better photocatalytic activity observed for the Ag(0.01)-doped Cd_0.1_Zn_0.9_S was proposed due to higher crystallinity, better absorption of visible light, and the formation of an optimum amount of Ag species, which facilitates the electron–hole separation and increase the stability of the catalyst, and the formation of less Ag_2_S, which would suppress the reaction.

## Experimental

**Preparation of Ag-doped Cd_0.1_Zn_0.9_S.** Hydrothermal and co-precipitation methods were used to prepare the Ag-doped Cd_0.1_Zn_0.9_S samples, which were labeled as Ag(*x*)-doped Cd_0.1_Zn_0.9_S, with *x* showed the doping amount of Ag (*x* = 0.01, 0.03, or 0.05 mol). For the hydrothermal method, a series of Ag(*x*)-doped Cd_0.1_Zn_0.9_S samples was synthesized similarly to the previous studies [9,16]. In a typical synthesis for Ag(0.01)-doped Cd_0.1_Zn_0.9_S, AgNO_3_ (Unilab, 99.9%), Cd(NO_3_)_2_·4H_2_O (Aldrich, 98%), Zn(CH_3_COO)_2_·2H_2_O (GCE chemicals, 98%) and CH_3_CSNH_2_ (Merck, 99%) with molar ratios of 0.01:0.1:0.9:1 were dissolved in distilled water at room temperature. The solution was then transferred into an autoclave with an inner Teflon lining, sealed and heated in an oven at 433 K for 8 h. The solution was left to reach room temperature naturally. The precipitate was separated by using a centrifuge and washed by using distilled water. The sample was then dried at room temperature under vacuum conditions. For the co-precipitation method, the series of Ag(*x*)-doped Cd_0.1_Zn_0.9_S was prepared similarly to the previous studies [9,16]. Typically, to synthesize Ag(0.01)-doped Cd_0.1_Zn_0.9_S, Na_2_S·*x*H_2_O (QRëc, 98%) solution was added dropwise to an aqueous solution containing AgNO_3_ (Unilab, 99.9%), Cd(NO_3_)_2_·4H_2_O (Aldrich, 98%) and Zn(CH_3_COO)_2_·2H_2_O (GCE chemicals, 98%) with molar ratios of 0.01:0.1:0.9. The solution was stirred for 12 h at room temperature. The resulting precipitate was filtered and washed several times with distilled water. The product then was dried in air at 343 K for 12 h.

**Characterizations.** XRD patterns were obtained on an X-ray diffractometer (Bruker Advance D8) using Cu Kα radiation (40 kV, 40 mA). The morphologies and size of the samples were observed by using field emission scanning electron microscopy (FESEM) with JEOL JSM 6701F at an accelerating voltage of 2 kV with platinum coating prior to analysis. DR UV–vis spectra were recorded at room temperature using a UV–visible spectrometer (Perkin Elmer Lambda 900). BaSO_4_ was used as a reflectance standard.

**Photocatalytic testing.** As described in the previous studies [9,16], photocatalytic hydrogen evolution was performed in a closed side irradiated-Pyrex cell equipped with a water condenser to maintain the temperature constant during the reaction. A 500 W halogen lamp was used as the visible-light source. Hydrogen gas evolved was identified by an online system with thermal conductivity detector (TCD) gas chromatography (GC, Agilent 7890A) using Supelco 13X molecular sieves and argon carrier gas, which amount was measured by volumetric method. In all experiments, the powder sample (0.2 g) was dispersed by magnetic stirring in an aqueous solution (50 mL) containing 0.25 M Na_2_SO_3_ and 0.35 M Na_2_S as the sacrificial agents. Nitrogen gas was flushed through the reaction cell for 30 min before reaction to remove air. In order to check the photocatalytic stability, the sample was reused without washing or drying. Before another 5 h irradiation in the second run, the reactor containing the tested sample was purged with nitrogen gas for 30 min to ensure that there was no residual hydrogen in the reactor.
